# Effect of Temperature on the Physico-Chemical Properties of a Room Temperature Ionic Liquid (1-Methyl-3-pentylimidazolium Hexafluorophosphate) with Polyethylene Glycol Oligomer

**DOI:** 10.3390/ijms12042598

**Published:** 2011-04-18

**Authors:** Tzi-Yi Wu, Bor-Kuan Chen, Lin Hao, Yu-Chun Peng, I-Wen Sun

**Affiliations:** 1 Department of Materials Engineering, Kun Shan University, Tainan 71003, Taiwan; E-Mails: t718z@yahoo.com.tw (T.-Y.W); t0322627@seed.net.tw (L.H.); skyandmeg@yahoo.com.tw (Y.-C.P.); 2 Department of Chemistry, National Cheng Kung University, Tainan 70101, Taiwan; E-Mail: iwsun@mail.ncku.edu.tw

**Keywords:** ionic liquids, density, viscosity, refractive index, excess molar volume, surface tension

## Abstract

A systematic study of the effect of composition on the thermo-physical properties of the binary mixtures of 1-methyl-3-pentyl imidazolium hexafluorophosphate **[MPI][PF_6_]** with poly(ethylene glycol) (PEG) [M_w_ = 400] is presented. The excess molar volume, refractive index deviation, viscosity deviation, and surface tension deviation values were calculated from these experimental density, ρ, refractive index, *n*, viscosity, η, and surface tension, γ, over the whole concentration range, respectively. The excess molar volumes are negative and continue to become increasingly negative with increasing temperature; whereas the viscosity and surface tension deviation are negative and become less negative with increasing temperature. The surface thermodynamic functions, such as surface entropy, enthalpy, as well as standard molar entropy, Parachor, and molar enthalpy of vaporization for pure ionic liquid, have been derived from the temperature dependence of the surface tension values.

## 1. Introduction

Ionic liquids (ILs) are a group of organic salts that result from the combination of several organic cations and inorganic anions, and they may be liquid at room temperature. This led to the discovery of the first room temperature molten salt in 1914, which was composed of an ethylammonium cation and nitrate anion and had a melting point of 12 °C [1]. The chemical and physical properties of ILs are interesting for several reasons, such as their high thermal stability, high conductivity, low density, extremely low vapor pressure, large electrochemical window, and their non-aqueous and non-toxic nature [2–7]. These properties make ILs ideal for many applications including their use as reusable solvents in organic reactions, and as electrolytes in batteries and solar cells [8–13]. In order to use such valuable materials for different commercial applications, though, the information about the thermodynamic and thermophysical properties of ILs and mixtures with other compounds are essential [14]. These properties, namely: viscosity, density, activity coefficients, excess molar volume, and refractive index, along with their thermochemical behavior are essential for the efficient design of industrial equipments. Moreover, the study of the physical properties of mixtures with ILs and other solvents is important because mixtures may be more appropriate than pure IL in some applications. For instance, it has been found that water and ethanol both increases the electrical conductivity substantially and decreases the viscosity appreciably [15], which may assist in improving electrodeposition using ILs.

Liquid-liquid equilibria of some two-phase systems containing selected ILs and salts has been studied in recent years [16,17]. For instance, Zhang *et al.* [18] determined the physical properties of the binary system of 1-ethyl-3-methyl imidazolium tetrafluoroborate +H_2_O. Their results show that the densities and viscosities are strongly dependent on the water content and weakly dependent on the temperature. Zafarani-Moattar investigated volumetric properties of 1-butyl-3-methyl imidazolium based ionic liquids in water and organic solvents [19]. Tian *et al*. [20] reported the density and viscosity of mixtures consisting of methyl formate, methyl acetate, ethyl formate, and acetone with 1-butyl-3-methylimidazolium tetrafluoroborate ([Bmim][BF_4_]) IL over the entire composition range at 298.15 K. However, to our knowledge, few reports are available on the physical properties of the binary system {ILs + polymer solution}. The design of entirely liquid systems composed of only ILs and polymers, with a very low vapor pressure over a wide range of temperatures, may be of high interest for particular applications.

Poly(ethylene glycol) (PEG) refers to an oligomer or polymer of ethylene oxide. PEG of various molecular weights have been widely used in processes across many industrial sectors, as a result of being non-toxic, biodegradable, inexpensive, widely available, and with a very low volatility [21,22]. Low molecular weight PEG (M_w_ = 400) is liquid at room temperature, making it easy to combine with ILs, generate solvent systems, and thus use in advanced, environmentally friendly processes [22].

In an attempt to explore the nature of interactions occurring between the mixing components, we report here the density, viscosity, refractive index, and surface tension of the binary mixture PEG400 and 1-methyl-3-pentylimidazolium hexafluorophosphate **[MPI][PF_6_]** from 293.15 to 353.15 K at atmospheric pressure. The excess molar volumes $V_{\text{m}}^{\text{E}}$ and the viscosity deviations Δη were calculated and correlated with composition data using Redlich-Kister polynomials. Using the quasi-linear surface tension variation with temperature observed for the pure IL, the surface thermodynamic properties, such as surface entropy, surface enthalpy, Parachor, the standard molar entropy, and molar enthalpy of vaporization (Δ_l_^g^*H*_m_^o^) were estimated. The surface tension deviations Δγ of the binary system {IL + polymer} solution were also investigated.

## 2. Results and Discussion

### 2.1. Neat Components

**Figure 1.**
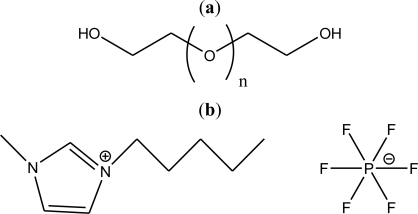
Chemical structure of (**a**) PEG(polyethyleneglycol), M_w_ = 400 and (**b**) 1-methyl-3-pentyl-imidazolium hexafluorophosphate (**[MPI][PF_6_]**).

The structure of PEG (polyethyleneglycol), M_w_ = 400 and 1-methyl-3-pentyl-imidazolium hexafluorophosphate (**[MPI][PF_6_]**) are shown in Figure 1. The thermophysical properties of neat IL **[MPI][PF_6_]** and PEG400 were measured from 301 to 359 K, and are presented in Table 1. In general, the density decreases with temperature for both neat substances, the correlation with temperature can be expressed using the following linear equation:

(1) ρ = A + BT

The characteristic parameters *A* and *B* were determined from the intercept and slope of the corresponding lines, and the best linear fitting *A* and *B* are listed in Table 2.

The viscosity in IL electrolytes is expected to vary significantly with temperature (lower viscosity at higher temperatures). As shown in Table 1, the viscosities of pure **[MPI][PF_6_]** and PEG400 decrease with increasing temperature due to the rise in the fluidity of the solution by increasing the kinetic energy of molecules. The viscosities and conductivities of pure **[MPI][PF_6_]** and PEG400 in the temperature range of 301 to 359 K were fitted using the Vogel–Tamman–Fulcher (VTF) equation [23]:

(2) $$\eta^{-1}\;=\;\frac{\eta_{o}}{\sqrt{T}}\text{exp}[\frac{-B}{\left(T\;-T_{o}\right)}]$$

where *T* is the absolute temperature and η_o_, *B*, and *T*_o_ are adjustable parameters. The best-fit η_o_ (cP), *B* (K), and *T*_o_ (K) parameters are given in Table 2. Neat **[MPI][PF_6_]** and PEG400 were fit very well by the VTF model over the temperature range studied.

The refractive index (*n*_D_) of neat IL **[MPI][PF_6_]** and PEG400 are 1.4141 and 1.4661 at 293.15 K, respectively, the former is comparable to a previously reported value for a similar hexafluorophosphate-based IL ([bmim][PF_6_], *n*_D_ = 1.40937 at 298.15 K) [24]. The molar volume (*V*_m_) of neat IL **[MPI][PF_6_]** was calculated from the molar mass (*M*) and experimental density (ρ) and using:

(3) $$V_{\text{m}}\;=\;\frac{M}{\rho }$$

The molar refraction (*R*_m_) of the liquid was calculated from experimental data of both molar volume (*V*_m_) and the refractive index (*n*_D_) at the studied temperatures using the Lorentz-Lorenz relation [25]:

(4) $$R_{\text{m}}\;=\frac{n_{\text{D}}^{2}\;-\;1}{n_{\text{D}}^{2}\;+\;2}\cdot V_{\text{m}}$$

The molar refraction of the neat ILs **[MPI][PF_6_]** and PEG400 are 55.1 and 96.5 at 293.15 K, respectively.

**Figure 2.**
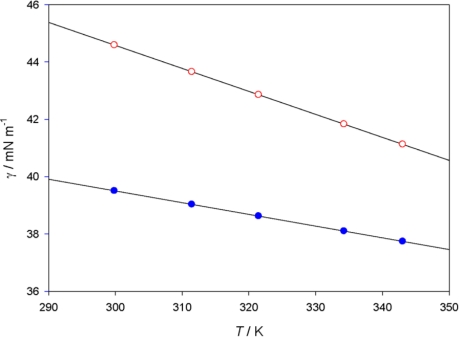
Plot of γ *versus T* of the **[MPI][PF_6_]** ( 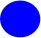) and PEG400 ( 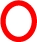).

The surface tension, γ, of PEG400 and **[MPI][PF_6_]** at various temperatures are shown in Figure 2, the surface tension of **[MPI][PF_6_]** at *T* = 308.15 K is 39.2 mN m^−1^, which is smaller than 1-butyl-3-methylimidazomethylimidazolium hexafluoro- phosphate, [BMIM][PF_6_] (γ = 43.8 mN m^−1^) [26], the lower surface tension of **[MPI][PF_6_]** is in agreement with the fact that it has the cation with the longer alkyl chain [27]. The surface tension of PEG400 is 43.8 mN m^−1^ at 309.05 K, which is larger than that of **[MPI][PF_6_]**. The surface tension, γ, of PEG400 and **[MPI][PF_6_]** linearly decreases with increasing temperature, according to the equation:

(5) $$\gamma \;=\;H^{\text{A}}\;-\;{TS}_{\text{o}}$$

(6) $$S_{o}\;=\;-{\left(\frac{\partial \gamma }{\partial T}\right)}_{p}$$

where the intercept, *H*^A^, can be identified with the surface enthalpy, and the slope, *S*_o_, can be calculated with the surface excess entropy, which is assumed to be temperature independent. The values of these parameters calculated for **[MPI][PF_6_]** are listed in Table 3 and compared with the IL values from other authors [28]. The estimated surface entropies are smaller than ethanol (0.086 mN m^−1^), water (0.138 mN m^−1^), benzene (0.13 mN m^−1^), and pyridine (0.1369 mN m^−1^) [29].

In terms of Glasser’s theory [30,31], the standard molar entropy, *S*^o^/J·K^−1^ mol^−1^, and the lattice energy, *U*_POT_/kJ mol^−1^, of the **[MPI][PF_6_]** at 298.15 K is calculated by following equations:

(7) $$S^{\text{o}}\;(298)/\text{J }\cdot \;\text{K }\cdot \;{\text{mol}}^{-1}\;=\;1246.5(V_{\text{m}}/nm^{3})\;+\;29.5$$

(8) $$U_{\text{POT}}/\text{k J }\cdot \;{\text{mol}}^{-1}\;=\;1981.2{\left(\rho /M\right)}^{1/3}\;+\;103.8$$

Accordingly, the standard molar entropy of **[MPI][PF_6_]**, *S*^o^(298 K)/J K^−1^ mol^−1^ = 487.4, and the lattice energy of **[MPI][PF_6_]**, *U*_POT_(298.15 K)/kJ mol^−1^ = 431.42, are obtained. In comparison with fused salts, the lattice energy of **[MPI][PF_6_]** is much lower than fused CsI (*U*_POT_ = 613 kJ mol^−1^at 298.15 K) [32], which has the smallest lattice energy among alkali halides.

The Parachor method, slightly modified by Sugden [33], has been used to calculate the Parachor, *P*, by the following equation:

(9) $$P\;=\;M_{\text{w}}\;\cdot \;\gamma^{\frac{1}{4}}/\rho$$

where *M*_w_ is the molar mass, γ is the surface tension, and ρ is the density. From this equation, *P* calculated for the **[MPI][PF_6_]** is 555.07 at 298.15 K, which is compared with *P* calculated from the other neutral compounds [34]. If *P* is known, it is possible to predict the surface tension and density of the ILs.

The value of the molar enthalpy of vaporization Δ_l_^g^*H*_m_^o^(298 K) of neat IL was estimated by Kabo’s empirical equation [35]:

(10) $$\Delta_{1}^{\text{g}}H_{\text{m}}^{\text{o}}\;(298\text{K})\;=\;A(\gamma V^{2/3}\;N^{1/3})\;+\;B$$

where *N* is Avogadro’s constant; *A* and *B* are empirical parameters; and their values are *A* = 0.01121 and *B* = 2.4 kJ mol^−1^, respectively. The molar enthalpy of vaporization for ionic liquid **[MPI][PF_6_]** calculated from Equation 10 was found to be 139.8 kJ mol^−1^ at 298.15 K.

### 2.2. Binary System

#### 2.2.1. Effect of Composition on Density and Excess Molar Volume

**Figure 3.**
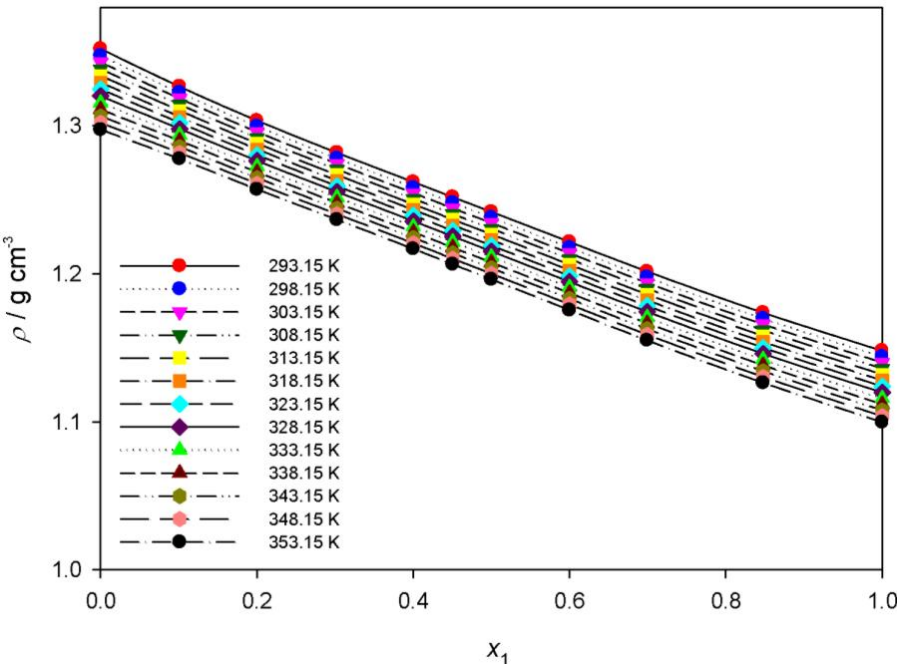
Density ρ of the {PEG400 (1) + **[MPI][PF_6_]** (2)} binary system as a function of temperature at various mole fractions. The lines represent the polynomial correlation.

**Figure 4.**
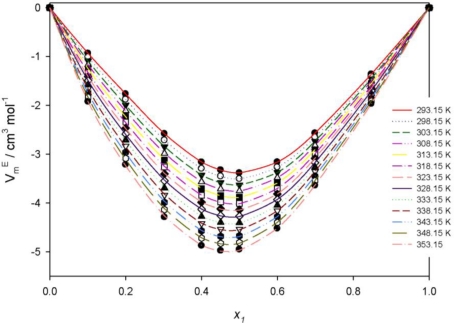
Excess molar volumes for the binary system {PEG400 (1) + **[MPI][PF_6_]** (2)} and fitted curves using the Redlich-Kister equation.

The densities of the binary mixture PEG400 + **[MPI][PF_6_]** were obtained as a function of PEG400 content at various temperature. As shown in Figure 3 and Table 4, the density decreases with temperature for the mixtures. The excess molar volume of the mixture, $V_{\text{m}}^{\text{E}}$, is a very sensitive thermodynamic property, indicating the existence of specific interactions and packing effects in the solutions. The excess molar volume $V_{\text{m}}^{\text{E}}$ was calculated from the experimental density values, using the following equation:

(11) $$V_{\text{m}}^{E}\;=\;\frac{x_{1}M_{1}\;+\;x_{2}M_{2}}{\rho }-\frac{x_{1}M_{1}}{\rho_{1}}-\frac{x_{2}M_{2}}{\rho_{2}}$$

where ρ_1_, ρ_2_, and ρ are the densities of PEG400, **[MPI][PF_6_]**, and their mixture, respectively; *M*_1_ and *M*_2_ are the molar masses of PEG400 and **[MPI][PF_6_]**, respectively. The calculated excess molar volumes for the present binary system are presented in Table 4 and shown in Figure 4. From the results obtained, it can be seen that the excess molar volume is negative with the maximum negative value approximately at *x*_1_ = 0.48, and the absolute values of the excess volume increase with increasing temperature. The negative value for the binary system is due to the fact that the interaction through hydrogen bonding between the imidazolium ring of **[MPI][PF_6_]** and the oxygen lone pair of PEG400 is strong and therefore has tightened the structure of the mixture; the filling effect of PEG in the interstices of ILs and the ion-dipole interactions between the PEG polar compound and the imidazolium ring of the ILs are also the contributors to the negative values of the molar excess volumes [36,37].

#### 2.2.2. Volume Expansivity and Excess Volume Expansivity

Based on the measured density values of this binary mixture, the excess molar volume $V_{\text{m}}^{\text{E}}$ and coefficient of thermal expansion α, can be calculated and correlated to characterize the influence of temperature and composition of the mixture on the properties. The density values as a function of the temperature can be used to calculate the thermal expansion coefficient or volume expansivity (α), using the following equation:

(12) $$\alpha \;=\;\frac{1}{V}\;{\left(\frac{\partial V}{\partial T}\right)}_{p}\;=\;-\frac{1}{\rho }{\left(\frac{\partial \rho }{\partial T}\right)}_{p}$$

where subscript *p* indicates constant pressure. The α values of neat **[MPI][PF_6_]** and PEG400, and their mixture are summarized in Table 5. The α values of ILs are in the range of 5.9 to 7.3 × 10^−4^ K^−1^, whilst the values of α for most molecular organic liquids are significantly higher (8 to 12 × 10^−4^ K^−1^). The thermal expansion coefficient of ILs is similar to those of water (α = 5.84 × 10^−4^ K^−1^ at 343.2 K) [38] and 1-methylimidazole (α = 8.63 × 10^−4^ K^−1^ at 298.2 K) [38].

The excess volume expansivity was calculated by the equation:

(13) $$\alpha^{\text{E}}\;=\;\alpha \;-\;\varphi_{1}^{\text{id}}\alpha_{1}\;-\;\varphi_{2}^{\text{id}}\alpha_{2}$$

where $\varphi_{1}^{\text{id}}$ is an ideal volume fraction given by the following relation:

(14) $$\varphi_{1}^{id}\;=\;\frac{x_{1}V_{\text{m1}}}{x_{1}V_{\text{m1}}\;+\;x_{2}V_{\text{m2}}}$$

in which *V*_mi_ stands for a molar volume of neat component i.

**Figure 5.**
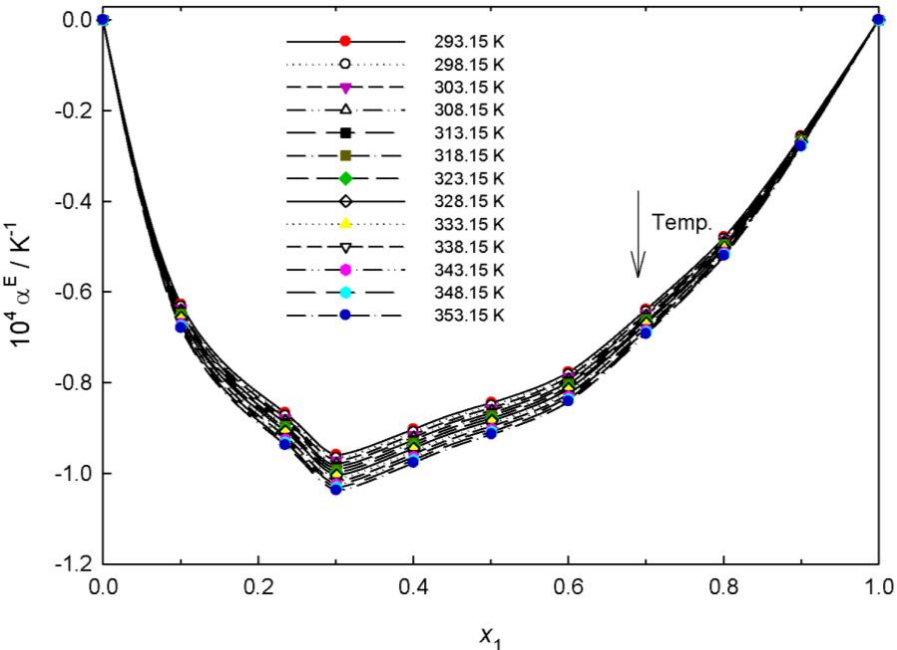
Plot of excess volume expansivity, α^E^, of the {PEG400 (1) + **[MPI][PF_6_]** (2)} binary system *versus* mole fraction *x*_1_ at various temperatures.

Typical concentration dependencies of excess expansivity are given in Figure 5 for the {PEG400 (1) + **[MPI][PF_6_]** (2)} binary system, the negative volume expansivity increase with increasing temperature is observed. The curves are asymmetrical, with the minimum located at PEG400 mole fraction about 0.3.

#### 2.2.3. Effect of Composition on Viscosity Deviation

From the experimental viscosities of the binary mixture, the viscosity deviations Δη (mPa·s) was defined as:

(15) $$\Delta \eta /(\text{mPa }\cdot \;\text{s})\;=\;\eta \;-\;x_{1}\eta_{1}\;-\;x_{2}\eta_{2}$$

where *x*_1_ and *x*_2_ are the mole fractions of PEG400 and **[MPI][PF_6_]**, respectively, and η, η_1_, and η_2_ are the experimental dynamic viscosities (mPa s) of the mixture, PEG400, and the IL, respectively. Experimental dynamic viscosity (η) and viscosity deviation (Δη) for the binary system studied are listed in Table 6.

**Figure 6.**
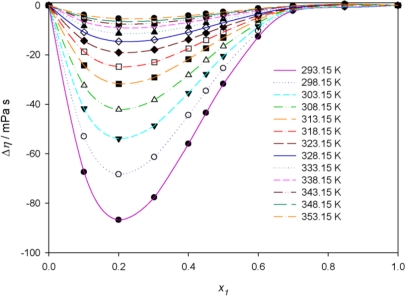
Viscosity deviations, Δη, *versus* the mole fraction at various temperatures for the binary mixture {PEG400 (1) + **[MPI][PF_6_]** (2)} and fitted curves using the Redlich-Kister equation.

The experimental viscosity deviations at various temperatures are plotted in Figure 6. The mixture of PEG400 with **[MPI][PF_6_]** shows negative deviations from ideality. The negative viscosity deviations decrease with increasing of temperature. This can be attributed to the specific interactions in mixtures, typically H-bonds, break-up as the temperature increases. The negative viscosity deviation reaches a maximum value at *x*_1_ = 0.2 (PEG400 mole fraction). The viscosity deviation depends on molecular interactions as well as on the size and shape of the molecules [39].

#### 2.2.4. Effect of Composition of Deviations in the Refractive Index

**Figure 7.**
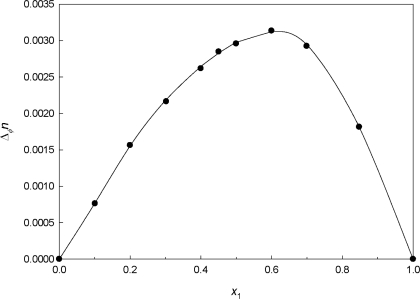
Deviation of Δ*_Φ_n* for the binary system {PEG400 (1) + **[MPI][PF_6_]** (2)} as a function of PEG400 mole fraction composition, *x*_1_, at 20 °C. The symbols represent experimental values, and the solid curves represent the values calculated from the Redlich-Kister equation.

Refractive indices *n* for all the {PEG400 + **[MPI][PF_6_]**}binary mixtures as a function of composition over the whole mole fraction range at *T* = 293.15 K are given in Table 7. Since deviation of *n* from ideality Δ*_Φ_n* correlates well with $V_{\text{m}}^{\text{E}}$ and physically interpretable as the deviation of reduced free volume from ideality when calculated on volume fraction basis [40] as:

(16) $$\Delta_{\Phi }n\;=\;n\;-\;\Phi_{1}n_{1}\;-\;\Phi_{2}n_{2}$$

where *Φ*_1_ and *Φ*_2_ are the volume fractions of component 1 (PEG400) and 2 (**[MPI][PF_6_]**), respectively. Values of *n*, *n*_D_^id^ (*n*_D_^id^ = *Φ*_1_*n*_1_ + *Φ*_2_*n*_2_), and Δ*_Φ_n* for the binary mixture are tabulated in Table 7. The Δ*_Φ_n* values for all the binary mixtures are plotted in Figure 7 as a function of volume fraction over the whole composition region. Δ*_Φ_n* values are asymmetric and positive over the entire composition range.

#### 2.2.5. Effect of Composition on the Deviations of Surface Tension

The surface tension deviations Δγ (mN m^−1^) were calculated from the following equation:

(17) $$\Delta \gamma /(\text{mN }\cdot \;{\text{m}}^{-1})\;=\;\gamma \;-\;x_{1}\gamma_{1}\;-\;x_{2}\gamma_{2}$$

where *x*_1_, *x*_2_ are the mole fractions of PEG400 and **[MPI][PF_6_]**, γ, γ_1_, and γ_2_ are the surface tension (mN m^−1^) of their mixtures, PEG400, and **[MPI][PF_6_]**, respectively.

**Figure 8.**
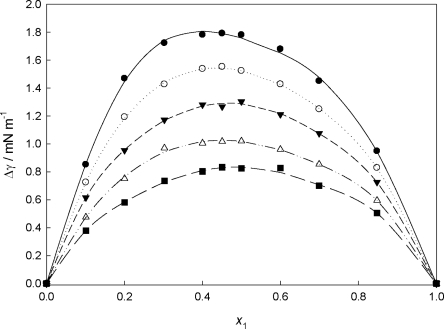
Surface tension deviation (Δγ) for the {PEG400 (1) + **[MPI][PF_6_]** (2)} system, as a function of the mole fraction, at different temperatures: (•) 298.15 K; (○) 308.15 K; (▾) 318.15 K; (Δ) 328.15 K; (▪) 338.15 K. Lines are fitting by the Redlich-Kister equation.

Figure 8 shows the dependence of the surface tension deviations as a function of the PEG400 mole fraction composition, *x*_1_, and temperature in the case of {PEG400 + **[MPI][PF_6_]**} binary mixtures. It can be seen that Δγ are positive over the entire composition range and decrease with increasing temperature. The positive values of the surface tension deviation may be considered as the interactions between like molecules (neat IL) are stronger than those unlike molecules (IL and PEG400 mixture) between the surface and the bulk region.

#### 2.2.6. Redlich-Kister Equation for Binary System

The binary excess property ( $V_{\text{m}}^{\text{E}}$) and deviations (Δη, Δ*_Φ_n*, and Δγ) at several temperatures were fitted to a Redlich-Kister-type equation [41]:

(18) $$\Delta Y\;(\text{or}Y^{E})\;=\;x_{1}(1\;-\;x_{1})\sum_{k=0}^{j}A_{i}{\left(1-2x_{1}\right)}^{i}$$

where Δ*Y* (*Y*^E^) represents $V_{\text{m}}^{\text{E}}$ (cm^3^ mol^−1^), Δη (mPa s), Δ*_Φ_n*, or Δγ (mN m^−1^); *x*_1_ denotes the mole fraction of PEG400, *A_i_* represents the polynomial coefficients, and *j* is the degree of the polynomial expansion. The correlated results for excess molar volumes ( $V_{\text{m}}^{\text{E}}$), viscosity deviations (Δη), refractive index deviations (Δ*_Φ_n*), surface tension deviations (Δγ), including the values of the fitting parameters *A_i_* together with the standard deviation σ, are given in Table 8, where the tabulated standard deviation σ [42] is defined as:

(19) $$\sigma ={\left[\frac{\sum {\left(\Delta Y_{\text{exp}}\;-\;\Delta Y_{\text{cal}}\right)}^{2}}{m-n}\right]}^{1/2}$$

where *m* is the number of experimental data points and *n* is the number of estimated parameters. The subscripts “exp” and “cal” denote the values of the experimental and calculated property, respectively. As shown in Table 8, the experimentally derived $V_{\text{m}}^{\text{E}}$, Δη, Δ*_Φ_n*, and Δγ values were correlated satisfactorily by the Redlich-Kister equation.

## 3. Experimental Section

### 3.1. Materials

1-methylimidazole (99%, Acros), 1-bromopentane (98%, Acros), and potassium hexafluorophosphate (99%, Showa) were obtained from commercial suppliers and used without further purification. Poly(ethylene glycol) [M_w_ = 400] was purchased from Showa Chemical Industry Co., Ltd, Japan.

### 3.2. Measurements

The density of the ionic liquids was measured gravimetrically with a 1 mL volumetric flask. Values of the density are ±0.0001 g mL^−1^. The viscosity (η) of the IL was measured using a calibrated modified Ostwald viscometer (Cannon-Fenske glass capillary viscometers, CFRU, 9721-A50). The viscometer capillary diameter was 1.2 mm measured by a caliper (model No. PD-153) with an accuracy of ±0.02 mm. The viscometer was placed in a thermostatic water bath (TV-4000, Tamson) whose temperature was regulated to within ±0.01 K. The flow time was measured using a stopwatch with a resolution of 0.01 s. For each IL, the experimental viscosity was obtained by averaging three to five flow time measurements. Measurements of the refractive index were conducted at 293.15 K with an ABBE refractive index instrument (Atago DR-A1), calibrated with deionized water with an accuracy greater than ±2 × 10^−4^. The water content of synthesized IL **[MPI][PF_6_]** was determined using the Karl-Fischer method; the content was below 100 ppm. The surface tension measurements were made by a Kyowa Interface Science’s automatic tensiometer CBVP-A3 (Japan). The uncertainty of the surface tension measurements is ±0.2 mN·m^−1^.

### 3.3. Synthetic Procedure of 1-Methyl-3-pentyl-imidazolium Hexafluorophosphate (**[MPI][PF_6_]**)

1-bromopentane (208 g, 1.38 mol) was added to a vigorously stirred solution of 1-methylimidazole (102.6 g, 1.25 mol) in toluene (125 mL) at 0 °C. The solution was heated to reflux at around 110 °C for 24 h, and then cooled to room temperature for 12 h. The toluene was decanted and the remaining viscous oil was washed with ether several times to yield a viscous liquid, which was dried *in vacuo* to give 1-pentyl-3-methylimidazolium bromide (**[MPI][Br]**) with a yield of approximately 82 %. ^1^H-NMR (300 MHz, D_2_O) δ: 8.65 (1H, s, NCHN), 7.41 (1H, m, CH_3_NCHCHN), 7.36 (1H, m, CH_3_NCHCHN), 4.12 (2H, t, NCH_2_(CH_2_)_3_CH_3_), 3.82 (3H, s, NCH_3_), 1.80 (2H, m, NCH_2_CH_2_CH_2_CH_2_CH_3_), 1.22 (4H, m, NCH_2_CH_2_CH_2_CH_2_CH_3_ and NCH_2_CH_2_ CH_2_CH_2_CH_3_), 0.79 (3H, t, N(CH_2_)_4_CH_3_). Elemental analysis is found (C, 46.26; H, 7.32; N, 11.97) and calculated (C, 46.36; H, 7.35; N, 12.02) for synthetic **[MPI][Br]**. KPF_6_ (0.32 mol) was added to a solution of **[MPI][Br]** (0.29 mol) in dichloromethane and stirred for 24 h. The suspension was filtered to remove the precipitated bromide salt. The organic phase was repeatedly washed with small volumes of water (around 30 cm^3^) until no precipitation of AgBr occurred in the aqueous phase upon the addition of a concentrated AgNO_3_ solution. The organic phase was then washed two more times with water to ensure the complete removal of the bromide salt. The solvent was removed *in vacuo* and the resulting IL was stirred with activated charcoal for 12 h. The IL was then passed through a short alumina column(s) (acidic and/or neutral) to give a colorless IL, which was dried at 100 °C *in vacuo* for 24 h or until no visible signs of water were present in the IR spectrum. Yields were 70 to 80 %. ^1^H-NMR (300 MHz, DMSO) δ: 9.02 (1H, s, NCHN), 7.70 (1H, m, CH_3_NCHCHN), 7.63 (1H, m, CH_3_NCHCHN), 4.12 (2H, t, NCH_2_(CH_2_)_3_CH_3_), 3.82 (3H, s, NCH_3_), 1.77 (2H, m, NCH_2_CH_2_CH_2_CH_2_CH_3_), 1.33–1.15 (2H, m, CH_2_CH_2_CH_2_CH_2_CH_3_ and CH_2_CH_2_CH_2_CH_2_CH_3_), 0.85 (3H, t, N(CH_2_)_4_CH_3_). Elemental analysis is found (C, 36.17; H, 5.71; N, 9.31) and calculated (C, 36.25; H, 5.75; N, 9.39) for synthetic **[MPI][PF_6_]**. The Br^-^ contents were confirmed with ICP-MS, being below 0.5% w/w.

## 4. Conclusions

Experimental density, dynamic viscosity, refractive index, and surface tension characterization for the binary system {PEG400 (1) + **[MPI][PF_6_]** (2)} were presented as a function of the temperature. The excess molar volume, excess volume expansivities, viscosity deviation, and surface tension deviation values, were calculated from these experimental data. The excess molar volume and excess volume expansivities are negative and continue to become increasingly negative with increasing temperature, whereas viscosity and surface tension deviation are negative and become less negative with increasing temperature. The refractive index was measured at 293.15 K for the binary system; the deviations of the refractive index have a positive value in the whole composition range. The fourth-order Redlich-Kister polynomial equation was applied successfully for the correlation of the excess molar volumes, viscosity deviation, refractive index deviation, and surface tension deviation, and the estimated coefficients and standard deviation values were also presented. The use of mixed ILs with poly(ethylene glycol) appears to be a promising approach for academic and industrial applications.

## Figures and Tables

**Table 1.** The viscosities and densities of neat **[MPI][PF_6_]** and PEG400 at various temperatures.

**[MPI][PF_6_]**		**PEG400**	
	
***T* (K)**	**η (cp)**	***T* (K)**	**η (cp)**
	
301.0	280.4	302.0	73.7
310.0	167.5	313.0	47.1
315.0	131.2	323.0	31.7
321.5	99.0	334.0	22.4
326.0	80.4	338.9	19.1
331.5	61.8	342.0	17.5
337.0	51.5	348.5	14.8
342.0	41.3	352.0	13.7
347.0	34.3	358.0	11.0
353.0	27.9		
357.9	26.0		
	
***T* (K)**	*ρ***(g cm^−3^)**	***T*(K)**	*ρ***(g cm^−3^)**
	
301.0	1.345	302.0	1.1415
310.0	1.336	313.0	1.1323
315.0	1.332	323.0	1.1244
321.5	1.327	334.0	1.1152
326.0	1.323	338.9	1.1113
331.5	1.318	342.0	1.1089
337.0	1.313	348.5	1.1037
342.0	1.308	352.0	1.1009
347.0	1.303	358.0	1.0966
353.0	1.298		
357.9	1.293		

**Table 2.** The adjustable parameters of density (ρ = *A* + *BT*) and the VTF equation parameters of viscosity ( $\eta^{-1}\;=\;\frac{\eta_{o}}{\sqrt{T}}\text{exp}[\frac{-B}{\left(T\;-\;T_{o}\right)}]$).

	**ρ**			**η**			
**Species**	*A*	10^4^*B*	*R*^2^^a^	η_o_ (mP s)	*T*_o_ (K)	*B* (K)	*R*^2^^a^
		
**[MPI][PF_6_]**	1.619	−9.111	0.9989	0.0512	155.7	1251	0.999
**PEG400**	1.384	−8.054	0.9998	0.0965	156.9	963.9	0.999

a Correlation coefficient.

**Table 3.** Surface thermodynamic functions *H*_A_ (Equation 5) and *S*_o_ (Equation 6) of the **[MPI][PF_6_]** and PEG400.

**Species**	***S*_o_ (mN m^−1^ K^−1^)**	***H*_A_(mN m^−1^)**
	
**[MPI][PF_6_]**	0.0409	51.77
**PEG400**	0.0802	68.63
**[BMIM]BF_4_**^a^	0.0593	61.80
**[BMPy]BF_4_**^a^	0.0607	63.1
**[BMIM]DCA**^a^	0.0775	71.88

a Reference [28].

**Table 4.** Experimental density (ρ) and excess molar volume (*V*_m_^E^) for the binary system {PEG400 (1) + **[MPI][PF_6_]** (2)}.

	***T*(K)**												
***x*_1_**	**293.15**	**298.15**	**303.15**	**308.15**	**313.15**	**318.15**	**323.15**	**328.15**	**333.15**	**338.15**	**343.15**	**348.15**	**353.15**
	**ρ (g cm^−3^)**												
0	1.3520	1.3475	1.3429	1.3384	1.3338	1.3293	1.3247	1.3202	1.3156	1.3110	1.3065	1.3019	1.2974
0.1010	1.3266	1.3225	1.3184	1.3144	1.3103	1.3062	1.3021	1.2980	1.2940	1.2899	1.2858	1.2817	1.2776
0.2002	1.3036	1.2997	1.2958	1.2919	1.2881	1.2842	1.2803	1.2764	1.2726	1.2687	1.2648	1.2609	1.2570
0.3024	1.2819	1.2781	1.2743	1.2705	1.2668	1.2630	1.2592	1.2554	1.2517	1.2479	1.2441	1.2403	1.2366
0.4002	1.2620	1.2583	1.2545	1.2507	1.2469	1.2432	1.2394	1.2356	1.2319	1.2281	1.2243	1.2205	1.2168
0.4507	1.2518	1.2480	1.2443	1.2405	1.2367	1.2329	1.2291	1.2253	1.2215	1.2178	1.2140	1.2102	1.2064
0.5000	1.2419	1.2381	1.2343	1.2305	1.2267	1.2228	1.2190	1.2152	1.2114	1.2076	1.2038	1.2000	1.1962
0.6000	1.2216	1.2177	1.2139	1.2100	1.2062	1.2023	1.1985	1.1947	1.1908	1.1870	1.1831	1.1793	1.1754
0.6994	1.2016	1.1977	1.1938	1.1899	1.1861	1.1822	1.1783	1.1744	1.1705	1.1666	1.1627	1.1588	1.1549
0.8476	1.1737	1.1698	1.1658	1.1619	1.1579	1.1540	1.1500	1.1461	1.1421	1.1382	1.1342	1.1303	1.1263
1	1.1480	1.1440	1.1400	1.1359	1.1319	1.1279	1.1239	1.1198	1.1158	1.1118	1.1077	1.1037	1.0997
	$V_{\text{m}}^{\text{E}}\;({\text{cm}}^{3}\;{\text{mol}}^{-1})$												
0	0.0000	0.0000	0.0000	0.0000	0.0000	0.0000	0.0000	0.0000	0.0000	0.0000	0.0000	0.0000	0.0000
0.1010	−0.9351	−1.0118	−1.0894	−1.1680	−1.2477	−1.3283	−1.4101	−1.4928	−1.5767	−1.6616	−1.7476	−1.8348	−1.9230
0.2002	−1.7666	−1.8793	−1.9935	−2.1090	−2.2260	−2.3445	−2.4646	−2.5861	−2.7091	−2.8338	−2.9600	−3.0879	−3.2173
0.3024	−2.5811	−2.7139	−2.8484	−2.9846	−3.1225	−3.2621	−3.4034	−3.5466	−3.6915	−3.8383	−3.9869	−4.1375	−4.2899
0.4002	−3.1625	−3.2954	−3.4301	−3.5664	−3.7044	−3.8442	−3.9857	−4.1291	−4.2742	−4.4211	−4.5700	−4.7207	−4.8733
0.4507	−3.3238	−3.4522	−3.5821	−3.7137	−3.8469	−3.9818	−4.1184	−4.2568	−4.3969	−4.5388	−4.6824	−4.8280	−4.9753
0.5000	−3.3847	−3.5064	−3.6297	−3.7544	−3.8808	−4.0087	−4.1383	−4.2695	−4.4024	−4.5370	−4.6733	−4.8113	−4.9511
0.6000	−3.1618	−3.2674	−3.3743	−3.4826	−3.5923	−3.7034	−3.8159	−3.9298	−4.0453	−4.1622	−4.2806	−4.4005	−4.5220
0.6994	−2.5687	−2.6519	−2.7361	−2.8213	−2.9077	−2.9952	−3.0839	−3.1737	−3.2646	−3.3568	−3.4501	−3.5447	−3.6405
0.8476	−1.3611	−1.4082	−1.4559	−1.5042	−1.5532	−1.6028	−1.6531	−1.7041	−1.7557	−1.8080	−1.8610	−1.9148	−1.9692
1	0.0000	0.0000	0.0000	0.0000	0.0000	0.0000	0.0000	0.0000	0.0000	0.0000	0.0000	0.0000	0.0000

**Table 5.** Experimental volume expansivity (α) and the excess volume expansivity (α^E^) for the binary system {PEG400 (1) + **[MPI][PF_6_]** (2)}.

	***T* (K)**												
***x*_1_**	**293.15**	**298.15**	**303.15**	**308.15**	**313.15**	**318.15**	**323.15**	**328.15**	**333.15**	**338.15**	**343.15**	**348.15**	**353.15**
	**10^−4^ α (K^−1^)**												
0	6.7387	6.7614	6.7844	6.8075	6.8307	6.8541	6.8777	6.9014	6.9253	6.9494	6.9736	6.9980	7.0226
0.1006	6.1525	6.1715	6.1906	6.2098	6.2292	6.2487	6.2682	6.2879	6.3078	6.3277	6.3478	6.3680	6.3884
0.2349	5.9498	5.9675	5.9854	6.0034	6.0214	6.0396	6.0579	6.0763	6.0948	6.1135	6.1322	6.1511	6.1700
0.3010	5.8914	5.9088	5.9263	5.9439	5.9616	5.9794	5.9974	6.0154	6.0335	6.0518	6.0702	6.0886	6.1072
0.4007	5.9776	5.9956	6.0136	6.0317	6.0500	6.0683	6.0868	6.1054	6.1241	6.1429	6.1618	6.1808	6.2000
0.5013	6.0503	6.0687	6.0872	6.1057	6.1244	6.1432	6.1622	6.1812	6.2004	6.2197	6.2391	6.2586	6.2782
0.6002	6.1310	6.1499	6.1688	6.1879	6.2071	6.2265	6.2459	6.2655	6.2852	6.3050	6.3249	6.3450	6.3652
0.7003	6.2944	6.3142	6.3342	6.3544	6.3746	6.3950	6.4155	6.4362	6.4569	6.4779	6.4989	6.5201	6.5414
0.8010	6.4771	6.4981	6.5193	6.5406	6.5621	6.5837	6.6054	6.6273	6.6494	6.6715	6.6939	6.7164	6.7390
0.9000	6.7307	6.7535	6.7763	6.7994	6.8226	6.8459	6.8694	6.8931	6.9170	6.9410	6.9651	6.9895	7.0140
1.0000	7.0156	7.0403	7.0651	7.0902	7.1154	7.1408	7.1664	7.1922	7.2181	7.2443	7.2706	7.2971	7.3239
	**10^−4^ α^E^ (K^−1^)**												
0.0000	0.0000	0.0000	0.0000	0.0000	0.0000	0.0000	0.0000	0.0000	0.0000	0.0000	0.0000	0.0000	0.0000
0.1006	−0.6279	−0.6320	−0.6361	−0.6403	−0.6445	−0.6487	−0.6530	−0.6574	−0.6617	−0.6662	−0.6706	−0.6752	−0.6797
0.2349	−0.8674	−0.8729	−0.8786	−0.8843	−0.8900	−0.8958	−0.9017	−0.9076	−0.9136	−0.9196	−0.9257	−0.9318	−0.9380
0.3010	−0.9599	−0.9660	−0.9722	−0.9785	−0.9849	−0.9913	−0.9978	−1.0043	−1.0109	−1.0176	−1.0243	−1.0311	−1.0379
0.4007	−0.9031	−0.9090	−0.9149	−0.9209	−0.9269	−0.9330	−0.9391	−0.9453	−0.9516	−0.9579	−0.9643	−0.9708	−0.9773
0.5013	−0.8446	−0.8502	−0.8557	−0.8614	−0.8670	−0.8728	−0.8786	−0.8844	−0.8903	−0.8963	−0.9023	−0.9084	−0.9145
0.6002	−0.7772	−0.7823	−0.7875	−0.7927	−0.7980	−0.8033	−0.8087	−0.8141	−0.8196	−0.8251	−0.8307	−0.8363	−0.8420
0.7003	−0.6390	−0.6433	−0.6476	−0.6519	−0.6563	−0.6608	−0.6652	−0.6698	−0.6744	−0.6790	−0.6836	−0.6884	−0.6931
0.8010	−0.4792	−0.4825	−0.4858	−0.4891	−0.4924	−0.4958	−0.4992	−0.5027	−0.5062	−0.5097	−0.5133	−0.5169	−0.5205
0.9000	−0.2565	−0.2583	−0.2601	−0.2619	−0.2638	−0.2656	−0.2675	−0.2694	−0.2713	−0.2732	−0.2752	−0.2771	−0.2791
1.0000	0.0000	0.0000	0.0000	0.0000	0.0000	0.0000	0.0000	0.0000	0.0000	0.0000	0.0000	0.0000	0.0000

**Table 6.** Experimental dynamic viscosity (η) and viscosity deviation (Δη) for the binary {PEG400 (1) + **[MPI][PF_6_]** (2)}.

	***T* (K)**												
***x*_1_**	**293.15**	**298.15**	**303.15**	**308.15**	**313.15**	**318.15**	**323.15**	**328.15**	**333.15**	**338.15**	**343.15**	**348.15**	**353.15**
**η (mPa·s)**													
0	463.8	336.7	249.8	189.1	145.6	114.0	90.5	72.9	59.4	48.9	40.7	34.2	29.0
0.1010	360.8	258.6	190.1	143.3	111.4	87.6	70.3	57.4	47.3	39.3	32.8	27.5	23.5
0.2002	306.6	218.6	160.0	120.5	94.0	74.1	59.8	49.1	40.8	34.0	28.5	23.9	20.4
0.3024	279.8	200.2	147.0	110.9	86.5	68.1	55.0	45.1	37.5	31.4	26.3	22.1	18.9
0.4002	267.0	192.9	142.6	108.1	84.3	66.3	53.4	43.7	36.2	30.4	25.5	21.5	18.4
0.4507	261.8	190.1	141.3	107.4	83.7	65.9	53.0	43.3	35.9	30.1	25.3	21.4	18.3
0.5000	256.2	187.1	139.6	106.5	83.0	65.5	52.7	43.0	35.6	29.9	25.1	21.3	18.2
0.6000	240.1	177.3	133.6	102.8	80.4	63.7	51.4	41.9	34.8	29.2	24.7	21.0	18.1
0.6994	215.6	161.0	122.5	95.2	75.0	60.0	48.6	39.9	33.2	28.0	23.8	20.4	17.6
0.8476	165.0	125.5	97.3	77.0	61.8	50.3	41.4	34.5	29.0	24.6	21.1	18.2	15.9
1	112.2	88.4	70.6	57.1	46.7	38.6	32.2	27.1	23.1	19.7	17.0	14.8	12.9
**Δη (mPa·s)**													
0	0.0	0.0	0.0	0.0	0.0	0.0	0.0	0.0	0.0	0.0	0.0	0.0	0.0
0.1010	−67.4	−53.0	−41.7	−32.4	−24.3	−18.8	−14.3	−10.9	−8.4	−6.7	−5.5	−4.8	−3.9
0.2002	−86.8	−68.4	−53.9	−42.1	−31.8	−24.8	−19.1	−14.6	−11.3	−9.1	−7.5	−6.5	−5.4
0.3024	−77.7	−61.4	−48.6	−38.3	−29.1	−23.0	−17.9	−13.9	−10.9	−8.7	−7.3	−6.3	−5.3
0.4002	−56.0	−44.5	−35.5	−28.2	−21.8	−17.5	−13.8	−10.9	−8.6	−6.9	−5.7	−5.0	−4.2
0.4507	−43.5	−34.7	−27.8	−22.2	−17.3	−14.1	−11.2	−9.0	−7.1	−5.7	−4.7	−4.1	−3.5
0.5000	−31.8	−25.5	−20.6	−16.6	−13.1	−10.8	−8.7	−7.0	−5.6	−4.5	−3.7	−3.2	−2.7
0.6000	−12.7	−10.4	−8.7	−7.1	−5.9	−5.0	−4.2	−3.5	−2.8	−2.2	−1.8	−1.5	−1.3
0.6994	−2.3	−2.1	−1.9	−1.6	−1.5	−1.3	−1.1	−1.0	−0.8	−0.5	−0.4	−0.3	−0.2
0.8476	−0.8	−0.7	−0.6	−0.2	0.0	0.2	0.3	0.4	0.4	0.4	0.5	0.5	0.5
1	0.0	0.0	0.0	0.0	0.0	0.0	0.0	0.0	0.0	0.0	0.0	0.0	0.0

**Table 7.** The refractive index, ideal $n_{D}^{\text{id}}$, deviation from ideality Δ*_Φ_n* for the binary mixture of {PEG400 (1) + **[MPI][PF_6_]** (2)} at 293.15 K (*n* ± 0.0003).

***x*_1_**	***n***	$n_{D}^{id}$	**Δ*_Φ_n***
0.0000	1.4141	1.4141	0
0.1010	1.4227	1.4219	0.00076
0.2002	1.4304	1.4288	0.00156
0.3024	1.4374	1.4352	0.00216
0.4002	1.4434	1.4408	0.00262
0.4507	1.4463	1.4435	0.00285
0.5000	1.4489	1.4459	0.00296
0.6000	1.4538	1.4507	0.00313
0.6994	1.4579	1.4550	0.00292
0.8476	1.4626	1.4608	0.00181
1.0000	1.4661	1.4661	0

**Table 8.** Redlick–Kister fitting coefficients *A_k_* and the standard deviation σ of the *V*^E^, Δη and Δ*_Φ_n* for the binary mixture of {PEG400 (1) + **[MPI][PF_6_]** (2)} system.

***T/*K**	***A*_0_**	***A*_1_**	***A*_2_**	***A*_3_**	***A*_4_**	*σ*
***V*^E^ (cm^3^ mol^−1^)**						
293.15	−13.533	0.0247	9.3514	−0.1098	−6.6156	0.002215
298.15	−14.025	−0.2587	9.2842	−0.1244	−6.7963	0.002998
303.15	−14.524	−0.5452	9.2161	−0.1395	−6.9797	0.004207
308.15	−15.029	−0.8352	9.1471	−0.1551	−7.1658	0.005590
313.15	−15.540	−1.1284	9.0771	−0.1713	−7.3548	0.007056
318.15	−16.057	−1.4251	9.0062	−0.1881	−7.5466	0.008574
323.15	−16.581	−1.7253	8.9342	−0.2055	−7.7414	0.010130
328.15	−17.112	−2.0289	8.8613	−0.2235	−7.939	0.011717
333.15	−17.65	−2.3361	8.7874	−0.2421	−8.1397	0.013333
338.15	−18.194	−2.6469	8.7125	−0.2613	−8.3435	0.014974
343.15	−18.745	−2.9613	8.6365	−0.2821	−8.5503	0.016642
348.15	−19.304	−3.2794	8.5594	−0.3018	−8.7604	0.018330
353.15	−19.87	−3.6012	8.4814	−0.323	−8.9736	0.020044

**Δη (mPa s)**						
293.15	−127.31	−451.48	−400.09	−1.8112	1.59	0.003938
298.15	−101.91	−355.24	−311.57	−0.4463	−0.0828	0.002017
303.15	−82.323	−279.56	−241.04	−0.2917	0.0104	0.000998
308.15	−66.223	−219.95	−181.46	0.6478	0.1556	0.002372
313.15	−52.416	−165.75	−129.71	0.0567	0.0901	0.000574
318.15	−43.129	−130.22	−93.895	0.2766	−0.183	0.000659
323.15	−34.858	−100.68	−66.419	0.5493	−0.9634	0.000931
328.15	−28.135	−77.373	−46.652	0.078	−0.2221	0.000266
333.15	−22.427	−60.466	−33.824	0.1233	−0.2623	0.000283
338.15	−17.873	−49.029	−26.275	0.1843	0.0986	0.000755
343.15	−14.917	−41.115	−20.748	0.213	0.0925	0.000846
348.15	−12.901	−35.813	−16.995	0.0273	−0.1882	0.000292
353.15	−10.905	−30.339	−12.82	0.0725	−0.0632	0.000192

**Δ***_Φ_****n***						
293.15	0.0119	−0.0051	0.003	0.0034	−0.0073	0.000028

**Δγ (mN m**^−^**^1^)**						
299.85	7.0343	1.642	4.4189	−0.4598	−3.686	0.029312
311.45	6.0978	1.2106	1.8964	−0.6364	0.1312	0.010338
321.45	5.1483	0.5758	0.8036	−0.1841	1.8383	0.015525
334.25	4.0704	0.6489	1.0679	−0.8076	0.8691	0.017579
343.05	3.356	0.1029	0.0254	−0.3028	2.1447	0.016906
